# Enhancing Prehospital Decision-Making: Exploring User Needs and Design Considerations for Clinical Decision Support Systems

**DOI:** 10.21203/rs.3.rs-5206138/v1

**Published:** 2024-11-20

**Authors:** Enze Bai, Zhan Zhang, Yincao Xu, Xiao Luo, Kathleen Adelgais

**Affiliations:** Pace University; Pace University; Pace University; Oklahoma State University; University of Colorado

**Keywords:** clinical decision support system, emergency medical services, prehospital workflow, user-centered design

## Abstract

**Background:**

In prehospital emergency care, providers face significant challenges in making informed decisions due to factors such as limited cognitive support, high-stress environments, and lack of experience with certain patient conditions. Effective Clinical Decision Support Systems (CDSS) have great potential to alleviate these challenges. However, such systems have not yet been widely adopted in real-world practice and have found to cause workflow disruptions and usability issues. Therefore, it is critical to investigate how to design CDSS that meet the needs of prehospital providers while accounting for the unique characteristics of prehospital workflows.

**Methods:**

We conducted semi-structured interviews with 20 prehospital providers recruited from four emergency medical services (EMS) agencies in an urban area in the northeastern U.S. The interviews focused on the decision-making challenges faced by prehospital providers, their technological needs for decision support, and key considerations for the design and implementation of a CDSS that can seamlessly integrate into prehospital care workflows. The data were analyzed using content analysis to identify common themes.

**Results:**

Our qualitative study identified several challenges in prehospital decision-making, including limited access to diagnostic tools, insufficient experience with certain critical patient conditions, and a lack of cognitive support. Participants highlighted several desired features to make CDSS more effective in the dynamic, hands-busy, and cognitively demanding prehospital context, such as automatic prompts for possible patient conditions and treatment options, alerts for critical patient safety events, AI-powered medication identification, and easy retrieval of protocols and guidelines using voice commands. Key considerations for successful CDSS adoption included prioritizing alerts to reduce alert fatigue and workflow disruptions, facilitating real-time data collection and documentation to enable decision generation, and ensuring trust and accountability while preventing over-reliance when using CDSS.

**Conclusion:**

This study provides empirical insights into the challenges prehospital providers face and offers design recommendations for developing CDSS solutions that align with prehospital workflows.

## INTRODUCTION

Prehospital emergency care involves providing emergency medical services (EMS) to patients with critical or altered conditions outside the hospital and transporting them to the nearest or most appropriate medical facility [1]. In this setting, time is essence and every second counts. If prehospital providers are unable to quickly evaluate a patient’s condition and implement the correct intervention, the patient’s outcome can be significantly affected, potentially leading to permanent disability or death [2, 3]. Moreover, the work environment in prehospital emergency care is dynamic, complicated, unpredictable, and sometimes unsafe, which can stress providers and impair their ability to make the right decisions [2, 4]. Research also indicates that prehospital providers often have limited access to cognitive and medical support on the scene, and many lack sufficient training for managing complex patient conditions [5, 6]. All these factors pose significant challenges to decision-making during prehospital emergency care.

To address these challenges, a limited amount of prior research has explored the ability and potential of computerized clinical decision support systems (CDSS) in assisting prehospital providers in making evidence-based, data-driven decisions. This body of research suggests that the use of CDSS can enhance patient safety and reduce errors in prehospital care [7, 8]. For example, Hagiwara and colleagues [9, 10] reported that CDSS can improve guideline compliance during patient evaluations, while Yu et al. [11] demonstrated in their study that CDSS can increase decision-making efficiency during mass casualty incidents.

Despite the potential of CDSS in enhancing prehospital care, several studies have reported issues with the adoption and usability of these systems, citing workflow disruptions and design flaws that hinder practical use [2, 7, 12]. For instance, one study found that a CDSS designed to assist decision-making about whether to take older patients who had fallen to an emergency department was used with only 12% of eligible patients [12]. A poorly designed CDSS may fail to account for real-world clinical contexts, leading to issues such as alert fatigue, workflow interference, medical errors, and increased on-scene time [10]. To improve the design of CDSS for prehospital providers, it is crucial to gather in-depth insights from end users (e.g., prehospital providers) on how CDSS should be designed and what features are important for the system to be effective and useful, while considering the unique work practices in prehospital emergency care. However, research on these aspects remains limited.

To address these research gaps, we adopted a qualitative approach and conducted an interview study with 20 prehospital providers to gain a deeper understanding of their decision-making challenges, technological needs, and considerations for successful CDSS development and implementation. This study is part of a larger research project aimed at using user-centered design principles and methods to iteratively design, develop, and evaluate a CDSS that aligns with prehospital workflows and providers’ needs. Our study makes the following contributions: (1) Empirical insights into the unique and prominent decision-making challenges faced by prehospital providers and their technology needs for addressing these challenges. (2) Design recommendations for developing CDSS solutions that can take into consideration care providers’ needs and the unique prehospital workflows.

## METHODS

### Data Collection

We conducted semi-structured interviews lasting between 45 to 90 minutes with 20 prehospital providers. Providers were recruited from four EMS agencies, all part of the 9–1-1 system in an urban area in the northeastern U.S. Of these participants, seventeen were paramedics, and the remaining three were emergency medical technicians (EMTs). Participants’ years of experience ranged from 5 to 40 years, with two participants holding positions as EMS directors. The participant information is summarized in Table 1. The director at each agency assisted in distributing recruitment information to the entire team, and those interested in participating were instructed to contact the researcher directly via email. We conducted interviews until data saturation was reached, meaning no new insights were being generated.

The interviews were conducted via teleconferencing tools (e.g., Zoom) or phone, depending on participants’ preferences. The interview questions focused on various aspects of their work, including professional experience, job responsibilities, field data collection and integration practices, decision-making processes, and associated challenges. The questions were developed based on the research objectives and existing literature. Additionally, the researchers conducted pilot interviews with two senior prehospital providers, who served as consultants for the project, to ensure that the questions were logically organized, clear, and free from ambiguity. Based on feedback from the pilot interviews, the questions were slightly modified. These two pilot interviews were not included in the data analysis.

All interviews were audio-recorded and transcribed verbatim for analysis. At the beginning of each interview, participants’ rights were explained by the researchers. Participants were assured that their information and insights would be used solely for research purposes, and their participation would remain confidential throughout the study. They reviewed and signed a consent form to indicate their agreement to be recorded. Each participant was compensated with a $60 gift card. The study received approval from the Institutional Review Board (IRB) at the corresponding author’s university (OHRP IRB# 0004707).

### Data Analysis

We employed a content analysis approach to analyze the data [13]. This method is particularly useful when researchers have limited prior knowledge about the medical domain or the specific problem under study [2, 14]. The data analysis process involved several steps, as explained below.

First, the researchers (EB, ZZ, and YX) reviewed all transcripts to familiarize themselves with the data and gain a general understanding of the content. Then, two researchers (EB and YX) independently coded a small subset of transcripts (n = 4) using NVivo—a qualitative data analysis software. The analysis focused on identifying the challenges prehospital providers face in patient data collection, integration, and decision-making, as well as their technological needs and key considerations for implementing CDSS in the dynamic prehospital care workflow. Words, sentences, or paragraphs that carried significant meaning were selected as semantic units and assigned codes—short phrases summarizing the meaning of the semantic unit. The initial codes were then discussed among the research team to determine which codes to retain, merge, or discard. This step resulted in the development of a codebook that defined each code.

Once the codebook was finalized, the two researchers (EB and YX) used it to analyze the remaining transcripts independently, with each researcher coding eight transcripts. The third researcher (ZZ) reviewed all the analyses to ensure the integrity and validity of the data analysis. Any new codes that emerged during this process were added to the codebook. Disagreements were discussed and resolved in research meetings involving all the researchers (the authors of this paper).

Finally, affinity diagrams—hierarchical classification of data elements—were used to group the finalized codes and identify overarching themes [15]. The main themes were discussed among all key researchers and presented to the two consultants for validation (e.g., ensuring that the researchers’ interpretation of prehospital decision-making challenges accurately reflected real-world practices).

## RESULTS

This section first outlines the current practices and challenges in prehospital decision-making. We then present insights gathered from care providers on design ideas for a prehospital-specific CDSS tool and, finally, illustrate the key factors that should be considered for such tools to be useful and effective in fast-paced medical environments.

### Current Practices and Challenges in Prehospital Decision Making

Upon arrival at the scene, prehospital providers must quickly evaluate the patient’s condition and the surrounding environment to assess the urgency of the situation (e.g., minor injury versus potentially life-threatening circumstances). Critical decisions they need to make include deciding the appropriate treatment plan and protocol to follow, determining medication administration and dosage, and selecting the most suitable medical facility for patient transport. While decisions vary based on the specific patient scenario, we identified several common work practices as well as challenges in prehospital decision-making, which are detailed as follows.

First, as participants explained, although it is ideal to determine the underlying cause of certain symptoms (e.g., whether shortness of breath is caused by acute pulmonary edema, pneumonia, or asthma), reaching a definitive diagnosis is often not feasible in prehospital care. The primary reason is that they lack access to advanced equipment (e.g., computed tomography scanner) or lab tests required for an accurate diagnosis. Therefore, prehospital providers only rely on a combination of basic physical exams (e.g., listening to breath sounds), vital sign monitoring (which tracks a patient’s basic physiological functions), and their experience to rule out underlying conditions, rather than concluding with a definitive diagnosis. While not a perfect solution, this approach is often the most practical given the constraints of time, resources, and the dynamic field environment, as our participant explained: “*Every diagnostic test that we go through is designed to eliminate a possibility. Sometimes we don’t ever really get to the final diagnosis because we don’t have access to certain diagnostic testing. So, a lot of this is based off of physical exam and educated guesswork.*” *[P#1]*

Second, unlike some medical teams where the team leader (e.g., the physician in charge) makes all the decisions, decision-making in the prehospital setting is a collaborative activity that involves both intra- and inter-teamwork. At the intra-team level, team members often discuss patient examination findings, vital signs, and symptoms with each other to collectively make decisions that everyone agrees upon. At the inter-team level, it is common practice for prehospital providers to call online medical control (OLMC) or a remote emergency physician to receive medical guidance or advice regarding critical decisions, such as treatment plans and patient destinations. However, a significant challenge with getting remote expert support is the lengthy waiting time for connection, which often delays patient care, as one participant noted: “*A common problem that occurs is the long delay in actually speaking to the physician, sometimes because they are the only one available and are handling many different areas. They have to finish one call before getting to the next. Sometimes it can take 20 minutes, and that can be frustrating. Obviously, it delays patient care. The most prominent issue is that there is often a long wait to speak with a physician. What you’re supposed to do is just wait, because otherwise, you can get in trouble for acting without consulting the physician*.” *[P#2]*

Third, as many participants emphasized, decision-making is highly dependent on experience. However, even the most experienced providers may encounter unfamiliar situations and infrequent clinical conditions and may not immediately recognize the necessary treatments, let alone novice or less-experienced providers. For example, several participants mentioned that treating pediatric patients is particularly challenging and significantly different from managing adult patients. Because there are relatively fewer pediatric encounters compared to adults, many prehospital providers lack sufficient opportunities to practice their cognitive or psychomotor skills in the care of children, leading to limited experience and knowledge in managing acutely ill and injured children in the prehospital setting. Consequently, medical errors—such as incorrect medication dosages, improper equipment sizes, and workflow deviations—can occur during these low-frequency, high-stakes events, resulting in delayed care and suboptimal patient outcomes. As one participant explained: “*I don’t have all the tools that someone in the hospital has. We’re trying to figure out how to stabilize this patient, even though we probably haven’t encountered a case like this before.*” *[P20]*

Finally, in current prehospital care practice, CDSS tools are not readily available, as one participant noted: “*Comprehensive decision-making tools for conditions like stroke or sepsis are not yet fully integrated into many EMS systems, leaving a reliance on manual processes and clinician knowledge.*” *[P#17]* Several participants mentioned that they have been using subscription-based mobile applications to calculate medication dosages and search for medical protocols, even though they have to pay for these tools themselves. While these tools offer valuable support, they are only useful when prehospital providers already know which medications to administer or what symptoms the patient is exhibiting, mainly serving as a reference. The inability to automatically deliver decision support at the time of the decision making is a key limitation of these tools, as one participant noted: “*The difficulty lies in determining what treatment to give and like how intense for the treatment to be*. *While checking medication dosage is critical, it is not as critical as determining the right treatments*.” *[P#7]*

### User Needs and Suggested Design Ideas for Prehospital CDSS

Our participants suggested several design ideas for developing a prehospital-specific CDSS, including automatic prompts for possible patient conditions and treatment options, alerts for critical patient safety events, AI-powered medication identification, and easy retrieval of protocols and guidelines using voice commands. These ideas were mentioned and discussed extensively by multiple prehospital providers, indicating that they are the most needed and preferred CDSS features. We will describe each design idea in greater detail below.

### Prompts for Possible Patient Conditions and Treatment Options

As explained in the previous section, participants emphasized that their work focuses less on reaching a definitive diagnosis and more on considering and ruling out different patient conditions to narrow down the most likely cause of symptoms. Therefore, the support they need from a CDSS involves prompting them to consider various conditions and underlying causes. For example, if a patient is under one year old and their blood pressure is below 70 mmHg, prehospital providers should consider the possibility of ongoing hypotension—a sign of pediatric shock requiring rapid treatment and intervention. However, less experienced or newer providers might not recognize this critical condition, leading to treatment delays. Even experienced providers may overlook or fail to consider certain possibilities due to “tunnel vision”—a lack of awareness that can occur in medical decision-making when a care provider becomes overly focused on a single aspect of a patient’s care. Participants highlighted the usefulness of a CDSS that could alert them to consider possible patient conditions: “*A prompt. For instance, a female experiencing acute coronary syndromes might present with symptoms like nausea, which could be overlooked without a prompt flagging the condition as potentially serious. So, the CDSS could help us recognize a condition, especially [for] patients that are not immediately recognized as high acuity.*” *[P#7]*

Furthermore, beyond alerting providers to possible underlying conditions, the CDSS was also expected to automatically suggest treatment options (e.g., administering medications) and protocols to follow, ensuring timely and accurate interventions in high-pressure scenarios: “*It would be great if the system was smart enough to recognize the situation and recommend protocols.*” *[P#10]* This capability was considered valuable because prehospital providers may not have time to manually search for the appropriate treatments or guidelines. By prompting providers with advice for medication administration or specific protocols, the system could support real-time decision-making and reduce the cognitive load on providers.

### Alerts for Critical Patient Safety Events

Participants expressed concerns about being overwhelmed by too many alerts, which could disrupt their workflow. However, nearly all participants agreed that alerts for critical safety events—those that could lead to severe consequences—are essential. For example, alerts would be particularly useful when administering medications, helping providers avoid giving the wrong medication or dosage: “*A critical alert is obviously necessary, like a reminder or an alert about maximum dosage for a particular medication for a particular medication*.” *[P#18]* Therefore, while minimizing distractions is important, alerts for critical patient safety events are invaluable and should be incorporated thoughtfully into CDSS tools to enhance patient safety.

Providers also emphasized that these critical alerts must be designed in a way that effectively grabs their attention, especially in high-stress, fast-paced environments. This could be achieved through visual signals such as flashing lights or auditory cues like sounds, which ensure providers are immediately aware of potential patient safety issues: “*It’s useful to have an alert that could save someone’s life. I want maybe a red light that would flash in the corner.*” *[P#10]*

### AI-powered Medication Identification

Prehospital providers need to identify, collect, and document the medications that patients are currently taking or have taken in the past to ensure they are fully aware of the patient’s medical history, which is critical for making informed treatment decisions. For example, if a patient is on anticoagulant medications, they may have an increased risk of internal bleeding, which should be taken into consideration when developing the treatment plan.

However, there are a wide variety of medications, and prehospital providers may not be able to recognize all of them or know what they are used for: “*There’s always new drugs. Unless you’re a pharmacist, it’s hard to keep track.*” *[P#20]* This challenge is exacerbated when patients are unable to provide the names of their medications or explain why they are taking them. For example, older patients often store their pills in containers without the original bottles, making it difficult to identify their medications: “*Our patient population is usually very old. They have these things out in pill containers, and they don’t necessarily keep the bottles once they’re empty. Or worse, they keep the bottles but use them for other pills. So, I don’t know what pills they are taking.*” *[P#4]*

Considering these commonly encountered issues in prehospital care, several participants envisioned the possibility of leveraging artificial intelligence (AI) and more specific computer vision (CV) techniques to quickly identify a patient’s medications and understand the conditions those medications are used for. The envisioned CDSS system would have the capability to scan labels on medication bottles, or even the pills themselves, to find relevant information: “*I have no idea what they’re taking. We wanted to have a pill identifier, which could recognize a pill with blue and white colors and a label like T742. So, scanning the bottle or the pill would be helpful.*” *[P#1]*

### Easy Retrieval of Protocol and Guidelines via Voice Interaction

Under extreme time pressure and when facing unfamiliar patient scenarios, prehospital providers may not be able to recall the correct actions or procedures for treating specific patients. As such, participants emphasized that the CDSS should allow for quick searches of protocols or guidelines. Of particular interest was the idea of using verbal, natural language to interact with the CDSS, inspired by recent advancements in AI technologies. This voice-based interaction could not only expedite information retrieval but also reduce the need for manual input, a key usability concern of handheld computing devices given the hands-busy nature of prehospital work and the risk of cross-contamination in prehospital care: “*Maybe having something where it can pull up either the protocol or an algorithm as you’re speaking to it in natural language.*” *[P#4]*

### Key Considerations for CDSS Development and Use in Prehospital Care

In this subsection, we present several major design and domain-specific considerations for CDSS to ensure their successful deployment and adoption in the prehospital context.

### Prioritizing Alerts to Avoid Workflow Disruptions and Alert Fatigue

Too many alerts, especially during critical situations, can overwhelm providers and lead to desensitization or the disregard of important notifications—a phenomenon known as alert fatigue. Given the dynamic and time-critical nature of the prehospital environment, what and how decision supports to present need to be carefully designed and evaluated to avoid alert fatigue. To address this issue, prehospital providers emphasized the importance of balancing the frequency and urgency of alerts, such as implementing a tiered alert system. In this system, only the most critical issues (e.g., incorrect medication dosages) would trigger an audio alert for immediate attention, while less urgent notifications would be presented visually without audio sound, minimizing distractions for the providers: “*I think that providers should only be prompted by the most critical issues with an alert that is obvious to catch your attention.*” *[P#7]*

Additionally, while audio alerts can be an effective way to capture immediate attention, they must be carefully designed to avoid disrupting the providers’ patient care activities or negatively affecting the patient. Given that prehospital providers are constantly in close proximity to their patients, it is crucial that alert sounds are designed to be non-intrusive and subtle (e.g., similar to the notification sound of a smartphone when a new message arrives). Loud or jarring sounds could distract providers during critical care activities or even cause stress to patients, who might already be in a vulnerable state. As one provider noted: “*Alarms can scare patients, so I would prefer more subtle audio prompt of possible dosing error, rather than a loud buzz.*” *[P#20]* One participant even proposed using haptic feedback, such as device vibrations, instead of alert sounds to capture the provider’s attention: “*Could be alerted by haptic feedback like vibrations to ensure the provider’s attention is captured without disturbing the patient*.” *[P#18]* This method ensures that the necessary alerts gain the attention of prehospital providers without compromising the comfort and safety of the patients they are treating.

### Facilitating Real-time Data Collection and Recording for Decision Generation

When discussing the use of CDSS in real practice, many providers raised concerns about whether they could effectively use it if patient data is not collected and documented in real-time. Due to the fast-paced nature of their work, prehospital providers primarily focus on stabilizing patients and addressing life-threatening conditions, often leaving documentation until the end of their shift (e.g., after handing the patient over to the receiving care team at the hospital): “*We often don’t have time to document in real-time; we are too focused on patient care.*” *[P#6]*

Therefore, participants expressed a desire for more efficient data collection and documentation methods, which are crucial for implementing any decision support tools in hands-busy care settings. Techniques like speech recognition (e.g., using voice to dictate patient information, which can then be processed and analyzed by the CDSS) were considered useful options for achieving this goal: “*Can we leverage voice recognition to support or facilitate real-time data collection or documentation? At the very least, it could capture basic patient information, such as medications administered, treatments performed, and vital signs in real-time—stuff like that.*” *[P#4]*

Another critical consideration voiced by our participants is the seamless integration of CDSS with the EHR system and other devices currently used by prehospital providers (e.g., vital signs monitors). Participants noted that collecting and recording patient data into the EHR is already challenging and time-consuming. Therefore, the CDSS tool should leverage the data already recorded in the EHR, rather than requiring them to input the data again. Avoiding double documentation is essential, as one participant explained: “*We’re already using our tablets (the device for the EHR system), so I think it would be easier and more natural to integrate CDSS into our tablet.*” *[P#9]* Furthermore, if the CDSS is connected to vital signs monitors, it can provide more accurate and timely recommendations based on real-time patient vitals.

### Ensuring Trust, Accountability, and Professional Autonomy

While CDSS can greatly assist providers by offering diagnostic and treatment recommendations, they introduce complex challenges around trust, accountability, and professional autonomy. One of the primary concerns among participants is the fear of making a medical error based on flawed CDSS recommendations. In high-stakes medical environments, where rapid and accurate decision-making can mean the difference between life and death, the question of accountability becomes critical when a provider follows an inaccurate suggestion from the CDSS. Participants discussed who should be held accountable for such errors—the care provider or the system. This gray area presents a significant barrier to adoption: “*I don’t mind using the system as a tool, but I don’t want to feel like I have to follow its recommendations, especially if something feels off.*” *[P#20]*

Another significant concern among our participants is the potential over-reliance on CDSS, which could undermine care providers’ expertise. While CDSS tools offer valuable support, an overdependence on technology may erode the critical decision-making skills of trained professionals in the field. Participants emphasized the importance of maintaining high educational and training standards, staying current with medical practices, and, most importantly, retaining the final authority over their clinical decisions, rather than becoming overly dependent on CDSS for decision support, as explained by one participant: “*Wouldn’t it be better to have a trained provider on scene rather than solely relying on this decision support tool? The expectation for paramedics should be higher, to study and stay up to date.*” *[P#17]*

## DISCUSSION

In prehospital emergency care, split-second decisions can have a significant impact. In line with prior research [2], our study shows that prehospital providers face significant challenges in making effective decisions during high-stress, time-sensitive situations, especially when handling unfamiliar and complex patient conditions (e.g., a pediatric patient with altered mental status) [16, 17], or when lacking timely medical and cognitive support (e.g., guidance from a remote emergency care physician) [18]. Some providers chose to pay out of pocket for subscription-based mobile applications that offer cognitive support (e.g., checking protocols or calculating medication dosages), while others did not use such tools at all. This indicates that decision support in prehospital emergency care is not universally available and remains insufficient.

Prior studies have suggested the use of CDSS to guide providers in responding to critical patient conditions and reducing medical errors [9, 10, 19]. While these tools show promise, they also present limitations, which may result in low adoption rates [7]. Therefore, we sought to understand how CDSS should be designed to not only improve clinical practice but also achieve greater adoption among prehospital providers. To this end, we employed a user-centered approach by conducting interviews with prehospital providers. This study is part of a larger research effort aimed at designing, developing, and evaluating a user-friendly CDSS that accommodates the unique workflow and needs of prehospital care providers. In the following section, we will reflect on our findings and position them within existing literature to discuss design implications for creating CDSS tools for emergency care settings such as prehospital care.

### Automatic provision of decision support as part of the clinical workflow.

Prior research on the evaluation of CDSS for hospital critical care teams has identified several key features that can enhance the effectiveness and adoption of CDSS by care providers [20]. One critical recommendation is that CDSS should automatically provide decision support at the time and location of decision-making, rather than requiring providers to click through multiple screens or manually select protocols [7, 20]. If CDSS requires additional effort from providers or disrupts the usual workflow, it is likely to face resistance and low adoption rates, as noted by Porter et al. [12]. Our study similarly revealed that prehospital providers prefer a CDSS that proactively prompts them to consider various patient conditions and offers treatment advice. Therefore, in prehospital and other emergency care settings, CDSS should be designed to integrate seamlessly into the workflow and proactively provide decision support without adding to the cognitive burden of care providers.

### Enabling natural language interaction with CDSS tools.

A unique characteristic of prehospital care is its hands-on nature. Prehospital providers often perform manual tasks, and their gloves may become contaminated with patient blood or fluids, which limits their ability to use handheld computing devices such as EHR systems, where CDSS tools are typically integrated [21]. Therefore, as our participants emphasized, the interaction with CDSS should be easy, intuitive, and achievable through natural verbal commands. Technically, this user need can be met with few anticipated issues (e.g., misheard by the system due to ambient noise), given the advancements in voice recognition technology and its increasing deployment in medical settings (e.g., digital medical scribes) [22]. However, the challenge lies in the social implications. Providers may feel socially awkward when asking the CDSS to display a protocol or calculate a medication dosage in front of a patient. This social consideration should be factored into the design of interaction methods for CDSS tools, ensuring they are not only functional but also mindful of the provider-patient dynamic.

### Addressing challenges in EHR documentation to enable real-time decision support generation.

Most CDSS tools require prehospital providers to navigate through EHR systems to collect and document various patient data (e.g., medical history, medications, and vital signs) for decision support. However, due to time constraints, providers in emergency care settings often struggle to use EHR in real-time and frequently postpone documentation until after patient care activities are completed [21, 23]. Our study also confirmed this work practice among prehospital providers. However, it can result in inaccurate or delayed data collection, posing a significant barrier to the effective use of CDSS in time-critical settings [24]. For example, one study [12] found that prehospital providers often used CDSS retrospectively, not as decision support, but merely to document their assessments and care decisions.

To enable real-time decision support generation, it is essential to address the challenges related to EHR documentation in prehospital care. Recent research has begun to address this issue through advanced technologies, such as leveraging natural language processing and voice recognition to enable verbal dictation into EHRs [25, 26], and using wearable devices (e.g., wristband [27] and smart glasses [28]) to facilitate hands-free documentation, addressing the limitations of manual EHR input. Additionally, more seamless system interactions, such as importing data from physiological monitors into EHRs, can help generate real-time alerts based on vital signs [29]. We believe that these research efforts and technological advancements will help alleviate the major barriers to real-time data collection and documentation in the field, thereby improving the effectiveness of CDSS.

### Prioritizing alerts to avoid alert fatigue and workflow disruptions.

CDSS have been found to cause alert fatigue and workflow disruptions when providers are inundated with frequent or irrelevant alerts [30]. This constant stream of notifications can distract providers from critical patient care tasks, leading them to habitually override alerts, thereby reducing the effectiveness of the system. For prehospital providers operating in high-pressure, time-sensitive environments, the consequences of alert fatigue can be particularly severe. To mitigate this issue, CDSS should be designed to implement a tiered alert system, where only the most critical alerts demand immediate attention. For instance, alerts related to life-threatening conditions, such as incorrect medication dosages or rapidly deteriorating vital signs, should be presented prominently and with an auditory cue to ensure quick intervention. Meanwhile, less urgent notifications—such as minor guideline deviations—could be displayed visually without interrupting the provider’s workflow. This approach would allow prehospital providers to maintain their focus on immediate patient needs while still receiving necessary guidance from the system.

In addition, customizing alerts based on individual provider preferences and the specific context of the situation could further improve CDSS usability. By allowing providers to configure alert thresholds or turn off non-essential notifications in certain scenarios, CDSS tools can enhance user experience and prevent alert fatigue. We argue that a well-calibrated alert system will contribute to higher adoption rates of CDSS, as it supports decision-making without overwhelming users or disrupting their workflows. Future work can explore how to design a tiered alert system, which would require providers and policymakers to establish a comprehensive list of alerts categorized by varying levels of urgency and criticality.

### Leveraging the power of AI while enhancing their trust and accountability.

With advancements in AI techniques, traditional CDSS can now harness state-of-the-art algorithms (e.g., deep neural networks and knowledge graphs) to process vast datasets and generate highly patient-specific and contextually relevant recommendations at the point of care [31, 32]. Our participants expressed enthusiasm about the potential of AI to improve decision-making accuracy and efficiency in prehospital care. They highlighted several potential applications of AI-powered CDSS in their work, such as medication recognition and treatment suggestions. Implementing these features are technically feasible; for example, advanced AI models for medication recognition have been developed and tested in recent work [33, 34].

Despite the vast potential, AI-powered CDSS faces challenges related to user trust and accountability, as highlighted in prior literature [35, 36]. A major concern is: What happens if the system provides an incorrect recommendation and the provider follows it? Who is accountable for any resulting medical errors? Studies have suggested that increasing the transparency of AI reasoning and offering explanations to end users can help providers understand how a decision was made and evaluate whether the recommendation should be adopted in patient care [37, 38]. However, asking prehospital providers to review AI reasoning in time-critical situations is impractical and could lead to distractions and delays in care.

We propose addressing trust and accountability in AI-CDSS through three non-technical approaches. First, policies and regulations should be established before the widespread deployment of AI-CDSS in prehospital care, clearly defining provider accountability when using these systems [37]. Policymakers should take into consideration various aspects, especially care providers’ perspectives, to enact such policies and regulations which should encourage care providers to use AI-powered CDSS. Second, as noted in prior research [39], building trust in a new technology takes time, particularly in high-stakes tasks like decision-making. Providers should have the opportunity to learn the strengths and limitations of AI-CDSS and gain experience with the system through extensive testing and training before they are asked to use the system in real practice [40]. Third, a phased deployment approach can be potentially adopted so that a small group of care providers can test the use of AI-CDSS in real settings to identify unexpected issues and consequences as well as technical limitations. Those issues should be addressed in a timely, iteratively, and collaborative manner to make sure the AI-CDSS tool can seamlessly fit into the local context and integrate with existing computer systems [41]. Establishing mechanisms for continuous monitoring, assessment, and collecting feedback on the AI-CDSS is a key factor in determining the success of any health information technologies [42].

### Provision of training for effective adoption and use of CDSS.

For CDSS to be fully effective, comprehensive training programs are essential to ensure that prehospital providers can use these tools proficiently without diverting attention from patient care. Additionally, it is crucial that providers maintain their critical thinking and clinical judgment while using CDSS. Although CDSS can offer valuable support, over-reliance on these systems without a full understanding of their limitations can lead to negative outcomes, as both our participants and prior literature highlighted [38, 43]. Therefore, training should not only cover the technical aspects of CDSS operation but also focus on integrating these tools into the broader clinical decision-making process. In particular, training should emphasize that CDSS is a complementary tool, not a replacement for human decision-making. Prehospital providers must balance the use of technology with their own expertise, ensuring they retain decision-making autonomy even when using advanced tools like CDSS [38].

### Limitations of the Study.

Our study recruited prehospital providers from a single region—an urban area on the East Coast of the U.S.—which may limit the generalizability of the findings. The perspectives shared by these providers may not be representative of those in other regions of the U.S., particularly in rural or suburban settings where the resources, patient demographics, and organizational structures differ. Similarly, international differences in prehospital care systems, policies, and technology adoption could mean that providers in other countries have distinct attitudes and needs when it comes to CDSS adoption. Another limitation of our study is that most participants lacked direct experience with CDSS tools, which may have constrained their ability to envision how such systems could be used in their practice. Their insights into the features and design considerations for CDSS may have been shaped more by their current workflows and assumptions, rather than by firsthand knowledge of how CDSS could enhance or challenge their decision-making processes. Future research involving providers with CDSS experience could offer additional insights into the design and adoption of CDSS for emergency care providers.

## CONCLUSION

This study provides valuable insights into the design and development of effective CDSS to address the decision-making challenges faced by prehospital providers. For CDSS to be successful, it must seamlessly integrate into prehospital workflows, offering real-time support at the point of care. Essential features identified include prompts for possible patient conditions and treatment options, AI-powered medication identification, voice-command protocol retrieval, and prioritized alerts for critical patient safety events. While CDSS holds promise for enhancing decision-making and patient safety in prehospital emergency care, several key considerations—such as alert fatigue, lack of effective data collection mechanisms, and accountability—must be addressed to better align these systems with the unique and dynamic nature of prehospital workflows.

## Figures and Tables

**Table 1:** Participant Information

Participant ID	Role or Occupation	Years of Experience
#1	Paramedic	7 years
#2	Paramedic	28 years
#3	Paramedic	15 years
#4	Paramedic	12 years
#5	Paramedic and EMS Director	25 years
#6	Paramedic	18 years
#7	Paramedic	8 years
#8	Paramedic	30 years
#9	Paramedic and EMS Director	40 years
#10	EMT	20 years
#11	EMT	11 years
#12	Paramedic	23 years
#13	Paramedic	14 years
#14	Paramedic	13 years
#15	EMT	4 years
#16	Paramedic	21 years
#17	Paramedic	5 years
#18	Paramedic	14 years
#19	Paramedic	7 years
#20	Paramedic	10 years

## Data Availability

Anonymized participant quotes are included in the main manuscript. Study protocol and data dictionary (e.g., the codebook developed during data analysis) will be made available publicly upon acceptance. Due to IRB restrictions and the need to protect human subject confidentiality, the raw data such as audio recordings and transcripts cannot be shared publicly. However, the de-identified transcripts can be provided by the corresponding author upon reasonable request.

## Abbreviations

CDSS: Clinical Decision Support System
AI-CDSS: AI-powered Clinical Decision Support System
EMS: Emergency Medical Services
HER: Electronic Health Records
EMT: Emergency Medical Technicians
CV: Computer Vision
OLMC: Online Medical Control
